# Using Cellulose-*graft*-Poly(L-lactide) Copolymers as Effective Compatibilizers for the Preparation of Cellulose/Poly(L-lactide) Composites with Enhanced Interfacial Compatibility

**DOI:** 10.3390/polym14173449

**Published:** 2022-08-24

**Authors:** Fei Liu, Shan Lu, Weihong Cao, Juncheng Huang, Yi Sun, Yiting Xu, Meiling Chen, Haining Na, Jin Zhu

**Affiliations:** 1 Key Laboratory of Bio-Based Polymeric Materials Technology and Application of Zhejiang Province, Ningbo Institute of Materials Technology and Engineering, Chinese Academy of Sciences, 1219 Zhongguan West Road, Zhenhai, Ningbo 315201, China; 2 School of Food Science and Pharmaceutics, Zhejiang Ocean University, No. 1 Haida South Road, Lincheng Changzhi Island, Zhoushan 316000, China

**Keywords:** cellulose, poly(L-lactide), composite, compatibilizer, CO_2_-switchable solvent

## Abstract

Cellulose-*grafte*-poly(L-lactide) (C-*g*-PLLA) copolymers synthesized in a CO_2_-switchable solvent are proposed for use as effective compatibilizers for the preparation of cellulose–PLLA composites with enhanced interfacial compatibility. The effect of the molar substitution (*MS*_PLLA_) of the grafted PLLA side chain in the C-*g*-PLLA copolymer and the feeding amount of this copolymer on the mechanical and thermal properties and hydrophilicity of the composites was investigated. The composites had a largely increased impact strength with the incorporation of the compatibilizer. With the increasing of *MS*_PLLA_ and the feeding amount of the copolymer, the resulting composites had an increased impact strength. When 5 wt% C-*g*-PLLA with *MS*_PLLA_ of 4.46 was used as a compatibilizer, the obtained composite containing 20 wt% cellulose presented an impact strength equal to that obtained for the neat PLLA. The composites had a slightly decreased melting temperature and thermal decomposition temperature, but increased hydrophilicity due to the incorporation of the compatibilizer. This work suggests an effective method to improve the interfacial compatibility between cellulose and PLLA for the fabrication of fully bio-based composites with high performance.

## 1. Introduction

As one of the most important bio-based materials [1], poly(L-lactide) (PLLA) has attracted much attention due to its superior mechanical properties, strength, ease of processing, and good biocompatibility. However, the disadvantages of PLLA, such as its high cost, inherent brittleness, and poor crystallization ability, limit its industrial applicability. Currently, blending PLLA with low-cost renewable fillers is the preferred solution for the development of environmentally friendly composites with a low cost and superior properties [2]. As an important renewable filler for PLLA, cellulose has attracted much attention due to its high availability and low price [3]. However, hydrophilic cellulose contains a large number of polar hydroxyl groups, and there is obvious interfacial differences between it and the hydrophobic PLLA, resulting in poor compatibility between these two components [4]. By simply blending cellulose and PLLA together without improving the interfacial adhesion, the resulting composite has dramatically decreased mechanical properties compared to those of neat PLLA, especially with regard to impact strength [4,5,6,7]. Therefore, it is necessary to improve the interfacial compatibility between cellulose and PLLA by using effective compatibilizers.

In previous studies, a number of compatibilizers have been developed for this purpose. Generally, these compatibilizers can be categorized into two types, reactive and non-reactive compatibilizers. For the first type, compatibilizers containing functional groups, which can undergo reactions with cellulose or PLLA, such as anhydride [8,9,10,11,12,13], expoxy [14,15,16], isocyanate [17,18,19], silane [20,21], and imide [22], are used. The interfacial adhesion can be improved by the formation of chemical bonding. For the second type, the compatibilizers do not react with cellulose or PLLA, such as poly(ethylene glycol) (PEG) [23,24,25,26] and casein [27], and the interfacial adhesion can be enhanced by intermolecular interaction between the compatibilizer and cellulose or PLLA, such as hydrogen bonding [23,27]. Although the reactive compatibilizer is more effective than its non-reactive counterpart, it is still desirable to develop a highly effective non-reactive one to improve the compatibility of cellulose with PLLA.

Recently, modified cellulose derivatives such as cellulose esters (e.g., cellulose acetate, cellulose butyrate, and cellulose laurate) have been used as non-reactive compatibilizers. These amphiphilic cellulose derivatives are considered to have good miscibility with both cellulose and PLLA [28,29] due to the coexistence of a cellulose backbone and aliphatic acid side chain, and therefore are able to improve the compatibility of cellulose with PLLA. Inspired by these results, the PLLA chain was directly grafted to the cellulose backbone to obtain a cellulose-*graft*-PLLA copolymer (C-*g*-PLLA) [30], which could be a more effective non-reactive compatibilizer compared with those cellulose esters mentioned above, since this copolymer would have better miscibility with both cellulose and PLLA. Previous studies have explored the use of this copolymer for the modification of PLLA in order to improve the melt strength [31] and transparency [32] of the composites with only two components, i.e., PLLA and the modified cellulose. To the best of our knowledge, using this copolymer as a compatibilizer for the modification of a cellulose–PLLA blend (C/P) has not yet been studied.

Therefore, in this study, we proposed using C-*g*-PLLA as an effective non-reactive compatibilizer for the modification of a C/P blend in order to improve the interfacial compatibility between cellulose and PLLA. The thermal and mechanical properties and hydrophilicity of the resulting composites were systematically investigated, and the cross-sectional morphologies of the samples after tensile and drop weight impact characterization were characterized and analyzed. The influence of the molar substitution of the PLLA side chain and the amount of C-*g*-PLLA on the thermal and mechanical properties of the composites was studied.

## 2. Materials and Methods

Corncob cellulose was purchased from Shandong Shengquan Group Co. Ltd., dimethyl sulfoxide (DMSO, AR > 99%); N,N-dimethylformamide (DMF, AR > 99.9%), dichloromethane (DCM, AR > 99.5%), 1,8-diazabicyclo [5.4.0]-7-ene (DBU, AR > 99%), methanol (AR > 99.5%), and isopropanol (AR > 99.9%) were purchased from Aladdin Chemical (Shanghai) Reagent Co. Ltd.; poly(L-lactide) (PLLA, 4032D, *M*_n_ = 66 kDa, *M*_w_ = 178 kDa) was purchased from NatureWorks (Minnetonka, MN, USA); L-lactide (>98%) was purchased from TotalCorbion (Rayong, Thailand); carbon dioxide (CO_2_, >99.9%) was purchased from Ningbo Wanli Gas Company. All chemicals were used as received.

### 2.1. Synthesis of C-g-PLLA

C-*g*-PLLA was synthesized in a CO_2_-switchable solvent system according to our previous study [30]. In the first step, a homogeneous cellulose solution was obtained by dissolving cellulose in the CO_2_-switchable solvent. In the typical manner, DMSO (50.0 g), DBU (6.64 g), and corncob cellulose (2.36 g) were added to a 500 mL stainless steel reactor equipped with a gas inlet and outlet. The molar ratio of DBU to the anhydroglucose unit (AGU) in cellulose was 3:1. The reactor was closed and kept at 55 °C and 1 atm with mechanical stirring, and CO_2_ was continuously introduced for 2 h. A yellowish and transparent homogeneous cellulose solution with a concentration of 4 wt% was obtained.

In the second step, L-lactide was added to the cellulose solution in order to graft the PLLA chain to the cellulose backbone by ring-opening polymerization of L-lactide. As is carried out for a typical experiment, to the homogeneous cellulose solution (50.19 g), L-lactide (21.94 g) was added and the mixture was mechanically stirred for 12 h at 80 °C under a nitrogen atmosphere. The feeding molar ratio of the lactide unit (LAU) to AGU was adjusted to 12:1 or 14:1 in order to obtain C-*g*-PLLA with different molar substitution of the PLLA side chain (denoted as C-*g*-PLLA-12 and C-*g*-PLLA-14, respectively). When the reaction was completed, the mixture was cooled down to room temperature. The separation and purification of C-*g*-PLLA was carried out according to the protocol as reported in our previous work [30].

### 2.2. Preparation of C/P Blend and the Composites

The cellulose and PLLA were dried under reduced pressure at 80 °C for 12 h. Subsequently, PLLA, the C/P blend (weight ratio 20/80), and a series of composites (weight ratio 20/80/1, 20/80/3, 20/80/5) (denoted as C/P/12-1, C/P/12-3, C/P/12-5, and C/P/14-1, C/P/14-3, C/P/14-5 for blends with C-*g*-PLLA-12 and C-*g*-PLLA-14 as compatibilizers, respectively) were prepared by melt blending at 190 °C for 8 min with a laboratory-scale internal mixer at 50 rpm. The test samples were prepared by an injection molding machine (HTF90W, Ningbo, China). The injection pressure, temperature, and time were 3.0 MPa, 180–200 °C, and 30 s, respectively. The sample size was selected according to the test standards GB/T 1040.2-2006 (Beijing, China) and GB/T 9341-2008 (Beijing, China).

### 2.3. Characterization

Proton Nuclear Magnetic Resonance Analysis (^1^H NMR) was carried out at room temperature with DMSO-*d*6 as the solvent with an AVANCE III 400 MHz nuclear magnetic resonance spectrometer.

The thermal properties of the composite samples were determined using a NETZSCH DSC214 Differential Scanning Calorimeter. Under a nitrogen atmosphere, the samples were heated to 220 °C at a heating rate of 10 °C·min^−1^ and held for 5 min to eliminate thermal history. After that, they were cooled to 0 °C at a cooling rate of 10 °C·min^−1^ and held for 5 min, and then a second heating scan was performed from 0 to 220 °C at a heating rate of 10 °C·min^−1^. The glass transition temperature (*T*_g_), cold crystallization temperature (*T*_cc_), melting temperature (*T*_m_), crystallization enthalpy(Δ*H*_cc_), and melting enthalpy (Δ*H*_m_) of the samples were obtained from the second heating scans. The degree of crystallinity (*χ*_c_) of the samples was calculated according to Equation (1):

(1) $$\chi_{c}=\frac{\Delta H_{m}-\Delta H_{cc}}{\varphi \Delta H_{m}^{\theta }{}_{}}\times 100\%$$

in which $\Delta H_{m}^{\theta }$ is the melting enthalpy of complete crystallization of PLLA (93.6 J·g^−1^), and *φ* is the mass fraction of PLLA in the composite.

The stability of the samples was determined using a thermogravimetric analyzer (METTLER-TOLEDO TGA/DSC). Under a nitrogen atmosphere, the sample was heated from 50 to 800 °C at a heating rate of 20 °C·min^−1^. The temperature at which the weight loss was 5% was designated as the initial thermal decomposition temperature (*T*_5%_), and the temperature at the maximum derivative weight loss was designated as the maximum thermal decomposition temperature (*T*_d,max_).

The tensile properties were measured using a Zwick/Roell Z030 30KN universal material-testing machine, and the tensile speed was 10 mm·min^−1^. Five splines were tested in parallel for each sample, and the test results were averaged.

The drop weight impact properties of the blends were tested on a Zwick/Roell Amster HIT2000F with a sample size of L × W × H = 80 × 10 × 40 mm^3^ and a notch depth of 2.0 ± 0.2 mm with a V notch.

The water contact angle was measured with model OCA25 from the German Dataphysics company using 3μL droplets as an indicator. Each sample was tested 5 times, and the average value was taken as the final contact angle of the sample. The measurement was carried out using deionized water as the liquid by dropping water droplets on the sample.

Morphology analysis was conducted using a scanning electron microscope (SEM, Hitachi S4800, Tokyo, Japan). The test voltage was 8 kV, and the test current was 7 μA. Before the test, the samples were gold-sprayed to increase the electrical conductivity.

## 3. Results

### 3.1. Chemical Structure Analysis of C-g-PLLA

**Figure 1:**
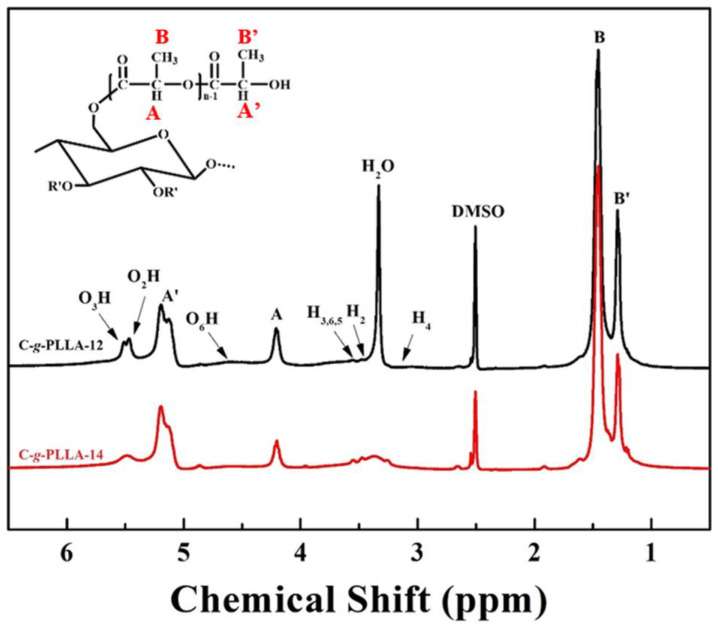
^1^H NMR spectra of C-*g*-PLLA.

Two C-*g*-PLLA samples (C-*g*-PLLA-12 and C-*g*-PLLA-14) with different molar substitutions of PLLA were synthesized. The feeding ratios of LAU:AGU were 12:1 and 14:1, respectively. The molecular structure of these two samples was characterized and confirmed by ^1^H NMR spectra, as shown in Figure 1.

The peaks at the chemical shift (*δ*) of 3.0–5.5 ppm are the signals of protons in O_2_*H*, O_3_*H*, O_6_*H*, *H*_2_, *H*_3, 6, 5_, and *H*_4_ in the cellulose backbone. The peaks at *δ* of 5.11 ppm (A′), 4.20 ppm (A), 1.29 ppm (B′), and 1.46ppm (B) can be assigned to the protons of methine protons of terminal lactyl, the methine protons of internal lactyl, methyl protons of terminal lactyl, and methyl protons of internal lactyl, respectively. The degree of polymerization (*DP*_PLLA_), degree of substitution (*DS*_PLLA_), molar substitution (*MS*_PLLA_), and weight content of PLLA (*W*_PLLA_) were then calculated according to Equations (2)–(5), respectively, and the results are listed in Table 1.

*DP*_PLLA_ = (*I*_B_ + *I*_B′_)/*I*_B′_ (2)

*DS*_PLLA_ = *I*_B′_/*I*_(O_2_*H* + O_3_*H* + O_6_*H*)_ (3)

*MS*_PLLA_ = *DP*_PLLA_ × *DS*_PLLA_ (4)

*W*_PLLA_ = 72*MS*/(162 + 72*MS*) (5)

where *I*_B_, *I*_B′_, *I*_O_2_*H*_, *I*_O_3_*H*_, and *I*_O_6_*H*_ are designated as the peak intensities of (B), (B′), (O_2_*H*), (O_3_*H*), and (O_6_*H*), respectively, and 72 and 162 are the molecular weights of the lactyl unit and AGU, respectively. The results show that the C-*g*-PLLA had relatively higher *DP*, *DS*, *MS*, and *W* for the grafted PLLA side chain when the feeding ratio of PLLA was increased.

### 3.2. Mechanical Properties

**Figure 2:**
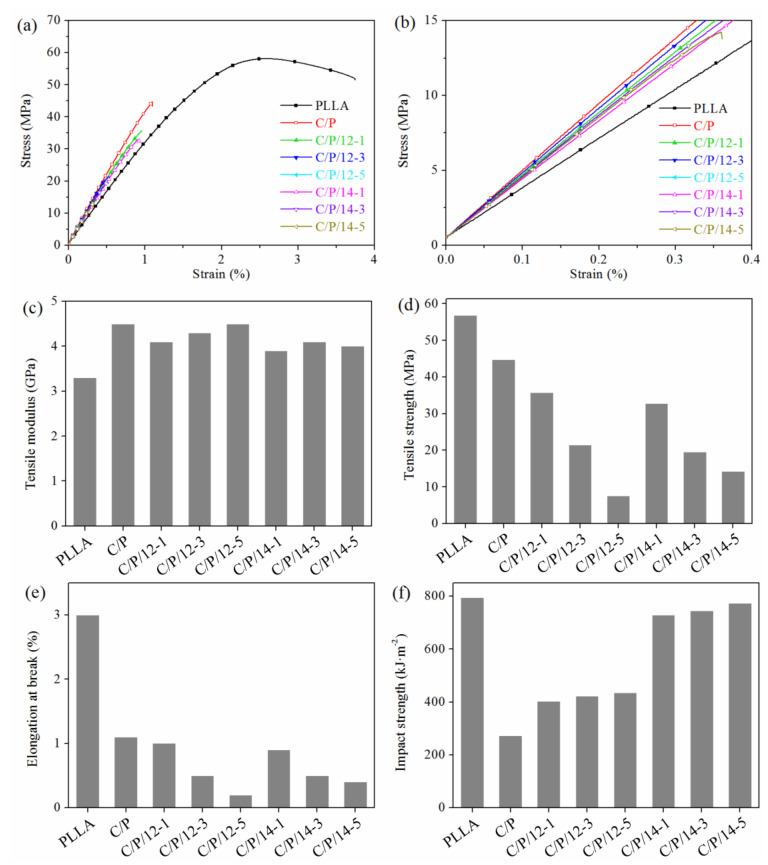
Mechanical properties of PLLA, C/P blend, and the composites: (**a**) representative tensile strain–stress curves, (**b**) tensile strain–stress curves in the range of 0–0.4% strain, (**c**) tensile modulus, (**d**) tensile strength, (**e**) elongation at break, and (**f**) notched impact strength.

The obtained C-*g*-PLLAs with different *MS*_PLLA_ were then used as compatibilizers for the modification of C/P blends. The weight ratio of the cellulose: PLLA was kept constant at 20:80, and the feeding amount of the compatibilizer was varied from 1 to 5 wt%. As a result, six composite samples were obtained. The mechanical properties of PLLA, the C/P blend, and the six composite samples were characterized by tensile and drop weight notched impact testing, and are shown in Figure 2.

It can be seen from the tensile strain–stress curves (Figure 2a,b) that the neat PLLA was a brittle material with a fairly high tensile modulus (3.3 GPa) and tensile strength (56.8 MPa), but low elongation at break (3.0%). After blending with 20 wt% cellulose, the obtained C/P blend had an increased tensile modulus of 4.5 GPa, but its tensile strength decreased to 44.7 MPa, and its elongation decreased at break to 1.1% (Figure 2c–e). These results are in good agreement with previous studies, as cellulose is commonly used as a renewable reinforcing filler for the modification of PLLA [17,33], which would increase the modulus, but decrease the elongation at break and strength of the product. After adding 1 wt% C-*g*-PLLA-12 as a compatibilizer, the resulting composite (sample C/P/12-1) had a slightly decreased tensile modulus (4.1 GPa), strength (35.7 MPa), and elongation at break (1.0%), compared with those of C/P. However, after further increasing the amount of C-*g*-PLLA-12 to 3 and 5 wt%, the resulting composites’ tensile moduli slightly increased to 4.3 and 4.5 GPa, but their tensile strength largely decreased to 21.5 and 7.5 MPa, and elongation at break largely decreased to 0.5 and 0.2%, respectively. This phenomenon was also observed when C-*g*-PLLA-14 was used as a compatibilizer, as shown in Figure 2c–e. Meanwhile, the compatibilizer with a longer PLLA side chain tended to result in composites with relatively lower tensile moduli and strength, which is probably due to the better miscibility resulting from the longer PLLA side chain [28,29].

The impact strength of neat PLLA was 793.9 kJ·m^−2^. However, the C/P blend with 20 wt% cellulose had a drastically decreased impact strength of 272.4 kJ·m^−2^. This result clearly reveals the poor interfacial adhesion and compatibility between cellulose and PLLA. Therefore, C-*g*-PLLA was introduced as a compatibilizer. When 1 wt% C-*g*-PLLA-12 was mixed with the C/P blend, the resulting composite had an obviously increased impact strength of 403.4 kJ·m^−2^, which gradually increased to 422.6 and 434.9 kJ·m^−2^ when increasing the amount of the compatibilizer to 3 and 5 wt%, respectively. These results strongly indicate that the interfacial adhesion between cellulose and PLLA was largely improved by C-*g*-PLLA. Moreover, after increasing molar substitution of the PLLA side chain in the C-*g*-PLLA compatibilizer, the resulting composites had further increased impact strength. Specifically, when 1 wt% C-*g*-PLLA-14 was used, the obtained composite had an impact strength as high as 727.9 kJ·m^−2^, which steadily increased to 745.4 and 772.3 kJ·m^−2^ when the amount of this compatibilizer increased to 3 and 5 wt%, respectively. These values are almost equal to those of neat PLLA, which unambiguously demonstrates that C-*g*-PLLA can be used as an effective non-reactive compatibilizer for the improvement of the interfacial adhesion and compatibility between cellulose and PLLA. It is even comparable to its reactive counterpart for the same purpose. For example, Dai et al. [4] synthesized epoxidized citric acid, which is used as a reactive compatibilizer for the microcrystalline cellulose–PLLA blend. The resulting composite containing 5 *wt*% of the reactive compatibilizer had approximately the same impact strength compared to that of neat PLLA.

### 3.3. Morphology Analysis

**Figure 3:**
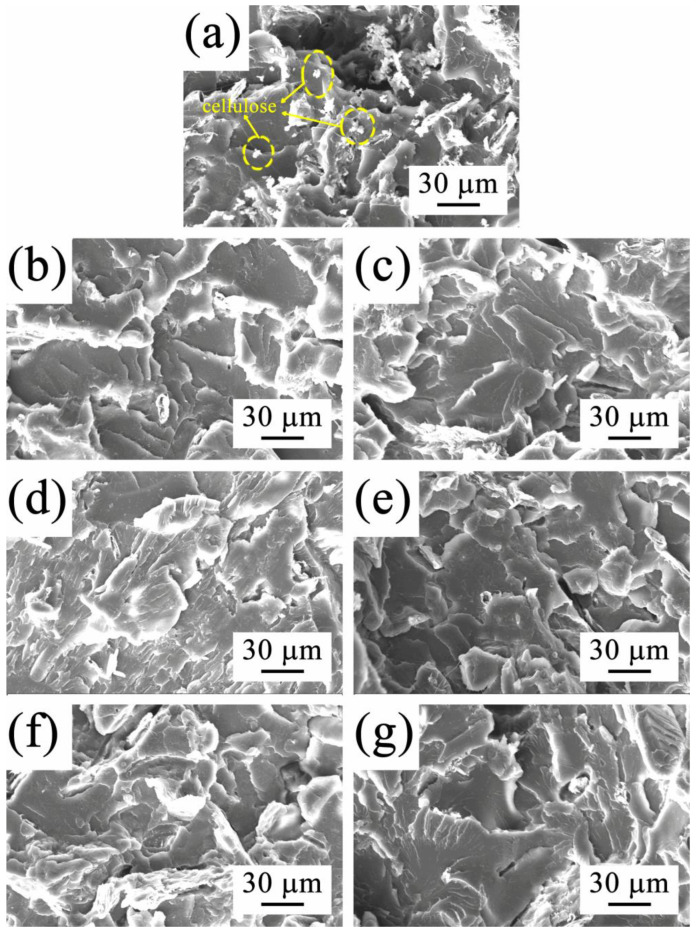
SEM images of cross-section after tensile testing: (**a**) C/P blend, (**b**) C/P/12-1, (**c**) C/P/12-3, (**d**) C/P/12-5, (**e**) C/P/14-1, (**f**) C/P/14-3, and (**g**) C/P/14-5.

**Figure 4:**
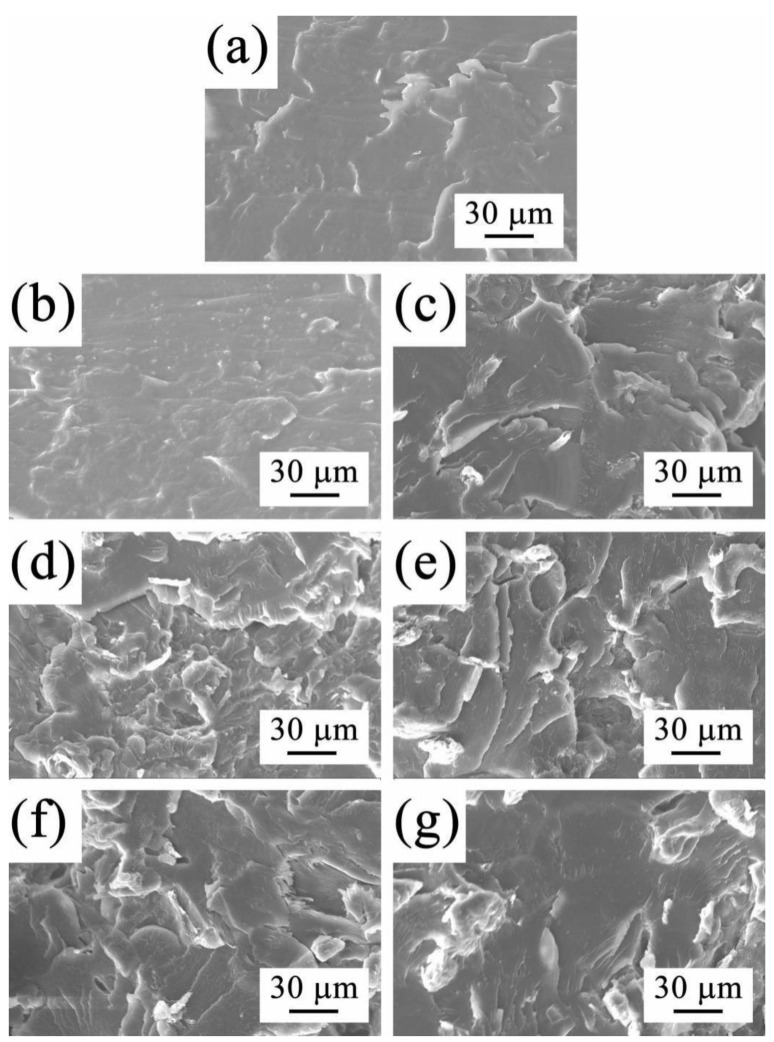
SEM images of cross-section after impact testing: (**a**) C/P blend, (**b**) C/P/12-1, (**c**) C/P/12-3, (**d**) C/P/12-5, (**e**) C/P/14-1, (**f**) C/P/14-3, and (**g**) C/P/14-5.

The compatibility between cellulose and PLLA in the C/P blend and the composites containing the C-*g*-PLLA compatibilizer was investigated by morphology analysis with SEM. The SEM images of the cross-section of the C/P blend and the composites after tensile and impact testing are displayed in Figure 3 and Figure 4, respectively.

It can be seen from Figure 3a that an aggregation of a large number of cellulose fillers were pulled out from the PLLA matrix after tensile testing, and there existed holes and gaps, indicating the poor interfacial adhesion and compatibility of the C/P blend without a compatibilizer. When C-*g*-PLLA was added, the morphologies of the cross-section of the composites after tensile testing were rough, and no holes or gaps could be observed (Figure 3b–g). Cellulose was well-dispersed and embedded in the PLLA matrix, and no aggregation of the filler was observed, showing good interfacial compatibility.

The cross-section of the C/P blend after impact testing was smooth and flat, as shown in Figure 4a, indicating its brittleness due to the poor interfacial adhesion. After adding C-*g*-PLLA as a compatibilizer, the morphologies of the cross-section of the composites became rough (Figure 4b–g), showing improved interfacial adhesion, which demonstrates that a greater amount of energy was absorbed during the impact testing.

### 3.4. Thermal Properties

**Figure 5:**
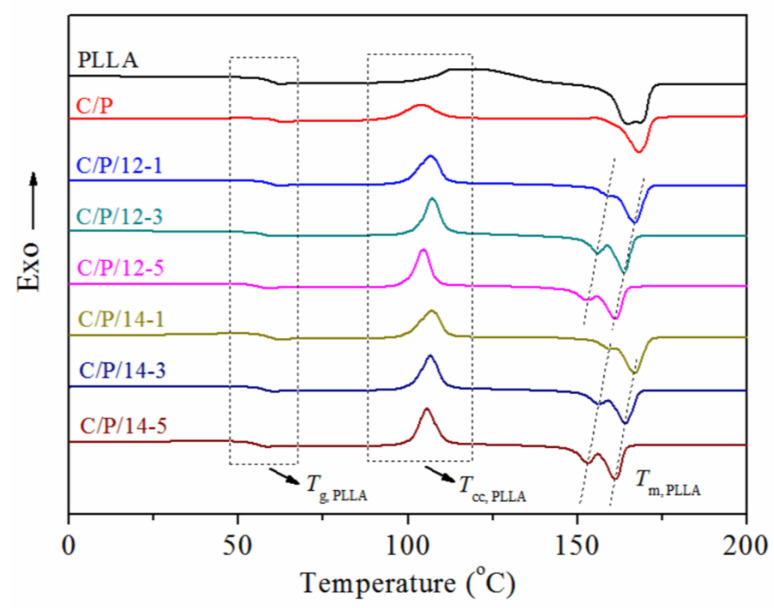
DSC curves for PLLA, C/P blend, and the composites obtained from the second heating scans.

DSC analysis was carried out to elucidate the influence of the compatibilizer on the glass transition temperature (*T*_g_) and melting temperature (*T*_m_) of the composites. The DSC curves from the second heating scans of PLLA, the C/P blend, and the composites are shown in Figure 5, and the results are listed in Table 2. Compared with the neat PLLA (*T*_g_ = 59.3 °C), the C/P blend had a slightly increased *T*_g_ (63.2 °C), which was also observed in a previous study [4]. Although C-*g*-PLLA had a relatively higher *T*_g_ (67.7 and 63.3 °C for C-*g*-PLLA-12 and C-*g*-PLLA-14, respectively) than neat PLLA, with the addition of C-*g*-PLLA as a compatibilizer, the obtained composites had a gradually decreasing *T*_g_ compared with that of the C/P blend. These results clearly indicate that the addition of the compatibilizer can benefit the local molecular movement of the PLLA matrix in the amorphous region, which results in the decreasing of the *T*_g_. Moreover, when the amount of C-*g*-PLLA was 5 wt%, the composites had an even lower *T*_g_ than that of neat PLLA, regardless of the molar substitution of the PLLA side chain on the cellulose backbone (58.5 and 58.1 °C for C/P/12-5 and C/P/14-5, respectively).

On the other hand, the addition of the compatibilizer also shows a certain influence on the crystalline region of the PLLA matrix in the composites [17,33]. When a compatibilizer was not added, the C/P blend had almost the same *T*_m_ and degree of crystallization (*c*_c_) as neat PLLA (Table 2), indicating poor intermolecular interaction between cellulose and PLLA. Although both of them showed only one *T*_m_ from the second heating scans in the DSC curves (Figure 5), the C/P blend showed an obvious cold crystallization (*T*_cc_) peak at 103.8 °C, which is probably because cellulose can play a role as a crystal-nucleating agent [32]. When C-*g*-PLLA was used as a compatibilizer, all composites showed an obvious *T*_cc_ and two *T*_m_. Firstly, all composites had a relatively higher *T*_cc_ than the C/P blend, which decreased with the increasing amount of the compatibilizer. This phenomenon can be ascribed to the increased miscibility of cellulose and PLLA due to the addition of C-*g*-PLLA as a compatibilizer [32]. Secondly, the main melting temperatures (*T*_m2,PLLA_) of the composites were lower than those of the neat PLLA and C/P blend, but the values of *c*_c_ were higher, and both *T*_m2,PLLA_ and *c*_c_ decreased with the increasing amount of the compatibilizer. These results indicate that although the addition of the compatibilizer is able to facilitate the crystallization of the PLLA matrix, it would result in a less perfect crystalline region, which gives rise to the decreasing of *T*_m_ and appearance of a second *T*_m_ at a relatively lower-temperature region.

**Figure 6:**
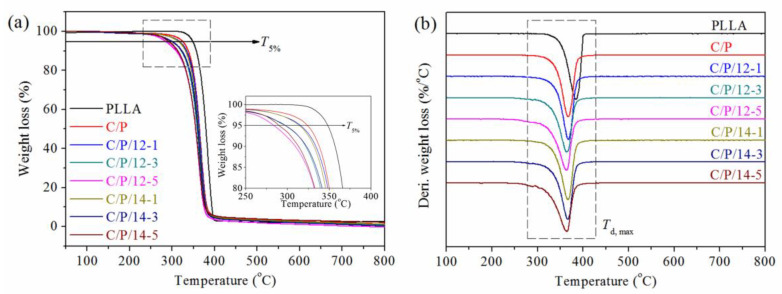
TGA (**a**) and DTG (**b**) curves of PLLA, C/P blend, and the composites.

The thermal stability of the C/P blend and the composites was evaluated by TGA analysis. The TGA and DTG curves are shown in Figure 6, and the correspondent initial thermal decomposition temperatures (*T*_5%_) and maximum thermal decomposition temperatures (*T*_d,max_) are listed in Table 2. It can be seen that *T*_5%_ and *T*_d,max_ decreased from 348.3 and 384.3 °C to 324.0 and 368.0 °C, respectively, with the addition of 20 wt% cellulose to the PLLA matrix. Furthermore, it is important to note that the use of a compatibilizer further decreased the *T*_5%_ of the composites, which gradually reduced with the increasing amount of the compatibilizer. The C/P/12-5 and C/P/14-5 composites had a *T*_5%_ of only 283.7 and 290.0 °C, respectively. Interestingly, the *T*_d,max_ remained almost unchanged for all composites, ranging from 364.0 to 367.3 °C, compared to that of the C/P blend, regardless of the type and amount of the compatibilizer used. These results indicate that the addition of the compatibilizer would accelerate the decomposition of the composites and reduce their thermal stability compared with neat PLLA. Nonetheless, they can still be processed safely without decomposition at temperatures below 250 °C, considering the processing temperature (e.g., 200 °C) for the composites is usually about 20–40 °C above the *T*_m_ of PLLA.

### 3.5. Hydrophilicity Characterization

**Figure 7:**
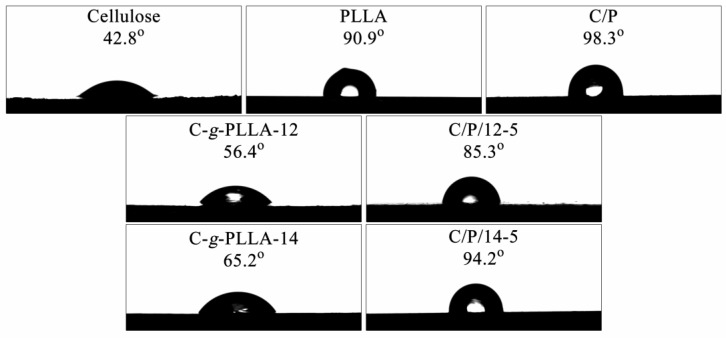
Static water contact angles of cellulose, PLLA, C/P, the compatibilizers, and the composites C/P/12-5 and C/P/14-5.

The hydrophilicity of cellulose, PLLA, the C/P blend, the compatibilizers, and the composites was characterized by measuring the static water contact angle (WCA). The results are shown in Figure 7. It can be seen that the cellulose was hydrophilic with a WCA of 42.8°, which is obviously lower than 90°, while neat PLLA was hydrophobic with a WCA slightly higher than 90°. When blending these two components together, the resulting C/P blend had a WCA of 98.3°, which is even higher than that of the neat PLLA, showing increased hydrophobicity. This result could be attributed to the poor interfacial compatibility between them. By grafting the hydrophobic PLLA side chain to the cellulose backbone, the resulting C-*g*-PLLA had a slightly increased WCA compared to the cellulose, indicating decreased hydrophilicity. The WCA increased to 56.4° and then to 65.2° with the increasing of PLLA molar substitution for C-*g*-PLLA-12 and C-*g*-PLLA-14, respectively. When 5 wt% of these two copolymers was used as a compatibilizer, the resulting composites’ WCA decreased to 85.3° and 94.2° for C/P/12-5 and C/P/14-5, respectively, compared to that of the C/P blend. These results clearly demonstrate that the use of a compatibilizer is able to enhance the interfacial compatibility between cellulose and PLLA, and therefore reduce the hydrophobicity of the composites.

## 4. Conclusions

In summary, this study proposed a simple and effective method to prepare a cellulose–PLLA composite with enhanced impact properties. Due to the good miscibility of the C-*g*-PLLA copolymer with both cellulose and PLLA, it can be used as an effective non-reactive compatibilizer for the preparation of a cellulose–PLLA composite with high performance. The addition of the compatibilizer effectively improved the impact strength and hydrophilicity, but decreased the *T*_g_, *T*_m_, and thermal stability of the composites, due to the increased interfacial adhesion and compatibility between cellulose and PLLA. The results show that the compatibilizer with a greater amount of PLLA side chain had more influence on the thermal and mechanical properties of the composite. By using only 5 wt% C-*g*-PLLA-14 as a non-reactive compatibilizer, the obtained composite containing 20 wt% cellulose had an impact strength as high as that of the neat PLLA, which is comparable to the effect of its reactive counterpart. Therefore, this study demonstrated the potential of using this cellulose derivative for the preparation of low-cost, high-performance fully bio-based composites from PLLA filled with large amounts of cellulose.

## Figures and Tables

**Table 1:** Chemical structure and glass transition temperature of C-*g*-PLLA.

Sample	*DP*_PLLA_	*DS*_PLLA_	*MS*_PLLA_	*W*_PLLA_ (%)	*T*_g_ (°C)
C-*g*-PLLA-12	2.76	1.43	3.95	63.7	67.7
C-*g*-PLLA-14	2.84	1.57	4.46	66.5	63.3

**Table 2:** Thermal properties of PLLA, C/P blend, and the composites.

Sample	*T*_g,PLLA_ (°C)	*T*_cc_,_PLLA_ (°C)	*T*_m1,PLLA_ (°C)	*T*_m2,PLLA_ (°C)	*ΔH*_cc_,_PLLA_ (J·g^−1^)	*ΔH*_m_,_PLLA_ (J·g^−1^)	*χ*_c,PLLA_ (%)	*T*_5%_ (°C)	*T*_d,max_ (°C)
PLLA	59.3	/	/	167.4	/	10.2	10.9	348.3	384.3
C/P	63.2	103.8	/	168.1	19.2	27.3	10.8	324.0	368.0
C/P/12-1	61.9	106.9	160.0	164.5	26.4	36.1	13.0	316.0	367.3
C/P/12-3	60.0	107.3	156.0	163.7	25.9	35.8	13.2	297.7	364.7
C/P/12-5	58.5	104.8	153.0	161.1	24.8	34.3	12.7	283.7	364.0
C/P/14-1	62.0	107.1	159.7	166.5	23.8	32.3	11.4	314.3	366.7
C/P/14-3	60.1	106.8	156.3	164.1	24.7	33.6	11.9	297.7	366.3
C/P/14-5	58.1	105.8	153.0	160.9	25.1	33.0	10.6	290.0	364.7

## Data Availability

Not applicable.
